# Supplementary material for: Identification of photoperiod-induced specific miRNAs in the adrenal glands of Sunite sheep (Ovis aries)
Source: Front Vet Sci. 2022 Jul 22;9:888207. doi: 10.3389/fvets.2022.888207 (PMC9354845; doi:10.3389/fvets.2022.888207)
Supplement: Supplementary file 1 [file Data_Sheet_1.ZIP › Supplementary file/miRDeep2 file.zip!/miRDeep2 file/Novel_Structure/SP42A2/Novel_587.pdf]

Provisional ID	:	NC_019466.2_20196
Score total	:	6.9
Score for star read(s)	:	3.9
Score for read counts	:	-0.3
Score for mfe	:	1.8
Score for randfold	:	1.6
Score for cons. seed	:	
Total read count	:	11
Mature read count	:	10
Loop read count	:	0
Star read count	:	1

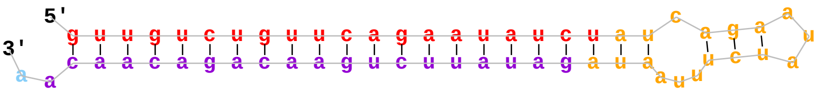

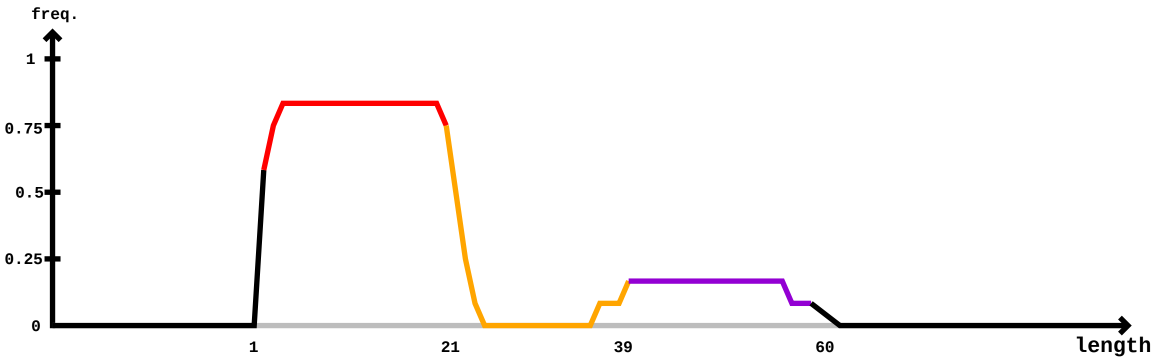


```
                          Mature                                  Star
5'- cuacauuugcuccaucauagguugucuguucagaauaucuaucagaauaucuuuaauagauauucugaacagacaacaaaaaacccaaguaaaugaagcuacuaauucau   -3'    obs
    cuacauuugcuccaucauagguugucuguucagaauaucuaucagaauaucuuuaauagauauucugaacagacaacaaaaaacccaaguaaaugaagcuacuaauucau          exp
    ((.((((((((.........((((((((((((((((((((((.(((...)))...))))))))))))))))))))))..........)))))))).))............   reads     mm      sample
    ....................guugucuguucagaauauc.......................................................................   1         0       seq
    ....................guugucuguucagaauaucu......................................................................   6         0       seq
    .....................uugucuguucagaauaucuau....................................................................   1         0       seq
    .....................uugucuguucagaauaucuauc...................................................................   1         0       seq
    ......................ugucuguucagaauaucuau....................................................................   1         0       seq
    .......................................................auagauauucugaacagaca...................................   1         0       seq
    ..........................................................gauauucugaacagacaaca................................   1         0       seq
```
